# Dietary Habits in the Offspring and Grandchildren of Long-lived Siblings

**DOI:** 10.1093/geroni/igaf122.2962

**Published:** 2025-12-31

**Authors:** Katrine Kristensen, Jacob Pedersen, Matthew Keys, Jytte Halkjær, Anne Tjønneland, Mary Wojczynski, Kaare Christensen, Christine Dalgård

**Affiliations:** University of Southern Denmark, Odense M, Syddanmark, Denmark; University of Southern Denmark, Odense M, Syddanmark, Denmark; University of Southern Denmark, Odense M, Syddanmark, Denmark; Danish Cancer Society, Copenhagen, Hovedstaden, Denmark; Danish Cancer Society, Copenhagen, Hovedstaden, Denmark; Washington University School of Medicine, St. Louis, MO, Missouri, United States; University of Southern Denmark, Odense M, Syddanmark, Denmark; Clinical Pharmacy, Pharmacology and Environmental Medicine, Odense M, Syddanmark, Denmark

## Abstract

Descendants of Danish longevity-enriched sibships demonstrate a general health and survival advantage throughout their lives. Diet may be one contributing mechanism. This study evaluates the dietary habits by estimating whether 203 descendants (median age 50, range 18-67, 58% females) of long-lived siblings in the Long Life Family Study adhered more to the official Danish dietary guidelines than 1015 age- and sex-matched population controls. From a comprehensive food frequency questionnaire (366 questions), participants’ adherence to dietary guidelines was calculated using a continuous score ranging from 0 (no adherence) to 6 (maximum). Data were collected from 2014 to 2018 (controls) and 2019 to 2023 (descendants). The median adherence score for male descendants was 4.2 (interquartile range 3.7-4.6) compared to 4.6 (4.1-4.9) for male controls, while the median score for female descendants was 4.9 (4.4-5.3), the same as for female controls. The adherence score for male descendants was 0.34 points lower than that for male controls (95% confidence interval -0.51;-0.18), with no significant difference for women (0.03, CI -0.11;0.16). When adjusting for birth year, the adherence score was 0.43 points lower for male descendants (CI -0.85;-0.02) and 0.10 points higher for women (CI -0.23;0.43). Descendents had a higher body mass index (men mean difference 0.7 kg/m2 (-0.2;1.6), women 0.6 kg/m2 (-0.3;1.6)), and fewer were current smokers (male descendants 15.1% vs 17.9% for the controls (p = 0.641); women 6.8% vs 13.8%(p = 0.053). In conclusion, the study found no evidence that descendants of long-lived siblings adhere more to the Danish dietary guidelines than a population-based control group.

